# Image-Guided Radiation Therapy Is Equally Effective for Basal and Squamous Cell Carcinoma

**DOI:** 10.3390/dermatopathology11040033

**Published:** 2024-11-19

**Authors:** Erin M. McClure, Clay J. Cockerell, Stephen Hammond, Evelyn S. Marienberg, Bobby N. Koneru, Jon Ward, Jeffrey B. Stricker

**Affiliations:** 1 University Hospitals Geauga Medical Center, University Hospitals Regional Hospitals, Chardon, OH 44024, USA; 2 Dermatology Residency Program, Dallas-Fort Worth/Lake Granbury, Granbury, TX 76048, USA; 3 Cockerell Dermatopathology, Dallas, TX 75235, USA; 4 Polaris Skin and Tissue Diagnostics, Myrtle Beach, SC 29572, USA; 5 Waccamaw Dermatology, Bypass, Suite 200, Myrtle Beach, SC 29588, USA; 6 Laserderm Dermatology, Smithtown, NY 11787, USA; 7 Summit Health/City MD, Rye, NY 10580, USA; 8 New York Radiation Oncology Associates, Jamaica, NY 11435, USA; 9 FHN Leonard C. Ferguson Cancer Center, Freeport, IL 61032, USA; 10 Stritch School of Medicine, Loyola University Chicago, Maywood, IL 60153, USA; 11 Paramount Oncology Group, Dubuque, IA 52001, USA; 12 Alabama College of Osteopathic Medicine, Dothan, AL 36303, USA; 13 Dermatology Solutions Group, Panama City, FL 32405, USA; 14 Dermatology Specialists of Alabama, Dothan, Dothan, AL 36303, USA

**Keywords:** non-melanoma skin cancer, image-guided superficial radiation therapy, freedom from recurrence, histology, basal cell carcinoma, squamous cell carcinoma, squamous cell carcinoma in situ

## Abstract

Non-melanoma skin cancers (NMSCs), including basal cell carcinoma (BCC) and squamous cell carcinoma (SCC), are highly prevalent and a significant cause of morbidity. Image-guided superficial radiation therapy (IGSRT) uses integrated high-resolution dermal ultrasound to improve lesion visualization, but it is unknown whether efficacy varies by histology. This large retrospective cohort study was conducted to determine the effect of tumor histology on freedom from recurrence in 20,069 biopsy-proven NMSC lesions treated with IGSRT, including 9928 BCCs (49.5%), 5294 SCCs (26.4%), 4648 SCCIS cases (23.2%), and 199 lesions with ≥2 NMSCs (1.0%). Freedom from recurrence at 2, 4, and 6 years was 99.60%, 99.45%, and 99.45% in BCC; 99.58%, 99.49%, and 99.49% in SCC; and 99.96%, 99.80%, and 99.80% in SCCIS. Freedom from recurrence at 2, 4, and 6 years following IGSRT did not differ significantly comparing BCC vs. non-BCC or SCC vs. non-SCC but were slightly lower among SCCIS vs. non-SCCIS (*p* = 0.002). There were no significant differences in freedom from recurrence when stratifying lesions by histologic subtype. This study demonstrates that there is no significant effect of histology on freedom from recurrence in IGSRT-treated NMSC except in SCCIS. These findings support IGSRT as a first-line therapeutic option for NMSC regardless of histology.

## 1. Introduction

Non-melanoma skin cancers (NMSCs), which include basal cell carcinoma (BCC) and squamous cell carcinoma (SCC) [1], constitute approximately one-third of malignancies diagnosed globally [2], are the most-diagnosed cancers in the United States [3], and are a cause of significant morbidity [4,5]. Incidences of BCC and SCC were 525 and 262 per 100,000 persons, respectively, in the US in 2019 [6], and NMSC incidence is thought to be increasing by around 2% annually [3,5,7]. Approximately 2000 people in the US and 65,000 globally die from NMSC each year [4,8,9]. NMSCs originate from epidermal cells via multifactorial pathogenesis, including exposure to ultraviolet and ionizing radiation, human papillomavirus, certain genetic diseases, and profound immune suppression, and are highly diverse in both clinical presentation and biological evolution [1].

**Figure 1:**
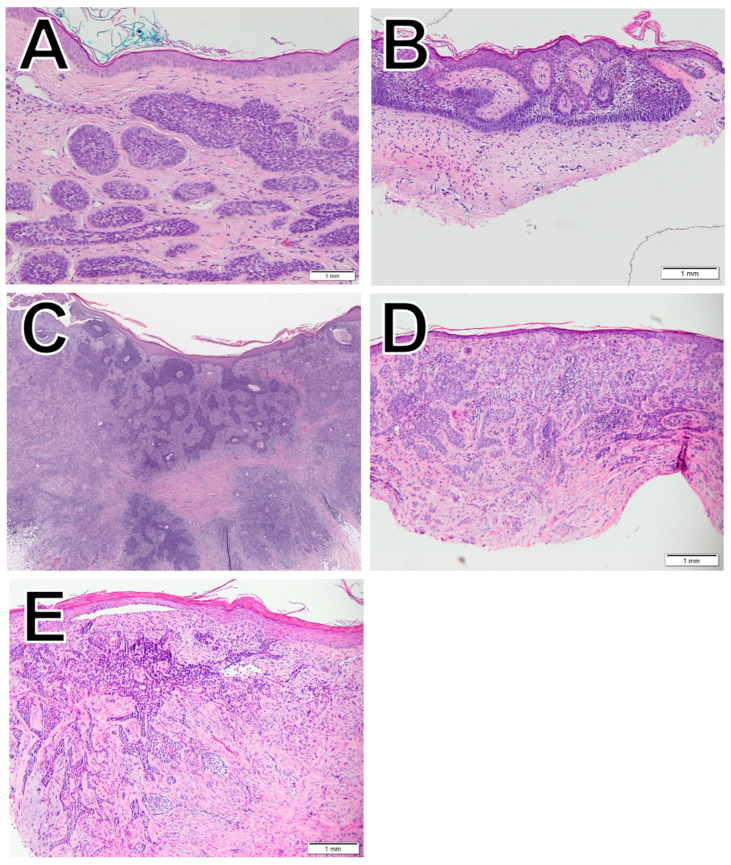
Histological examples of nodular BCC (**A**), superficial BCC (**B**), squamous differentiation BCC (**C**), infiltrative (**D**), and morpheaform BCC (**E**).

BCC (Figure 1), the most common type of skin cancer, originates in basal cells, which are at the base of the epidermis [10]. While it is rare for BCC to metastasize and mortality is therefore low, if left untreated, BCC can result in high morbidity via local invasion and tissue destruction [1]. There are over 25 morphological subtypes of BCC [11], and in this study, we evaluate five of them: nodular (Figure 1A), superficial (Figure 1B), squamous differentiation (Figure 1C), infiltrative (Figure 1D), and morpheaform (Figure 1E).

**Figure 2:**
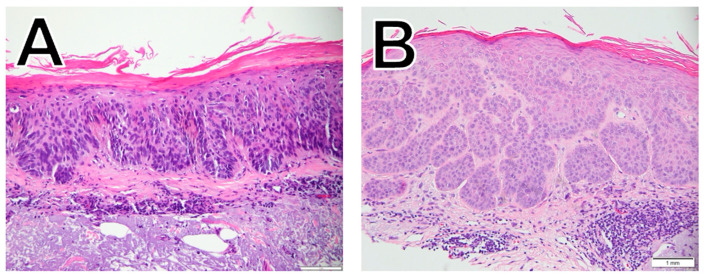
Histological examples of SCCIS (**A**) and well-differentiated SCC (**B**).

In contrast to BCC, SCC (Figure 2) originates in squamous cells, the flat cells in the superficial part of the epidermis. SCC subtypes range from slow-growing to aggressive, invasive tumors with a higher risk of metastasis [1,12]. The SCC subtypes included in this study are squamous cell carcinoma in situ (SCCIS; Figure 2A) and well-differentiated SCC (Figure 2B). SCC tumor characteristics such as site, thickness, and ability to spread and patient characteristics such as older age, male sex, prior treatment with B-Raf enzyme (BRAF) inhibitors, and concomitant immunosuppressive conditions are all associated with increased mortality [1]. SCCIS is the earliest form of SCC, with the cancer cells confined to the epidermis, and is not usually symptomatic but is characterized by large, red, scaly/crusted patches [10]. However, SCCIS can progress to invasive SCC, and early treatment is therefore recommended [10].

Current National Comprehensive Cancer Network guidelines for the treatment of localized, high- and low-risk BCCs and SCCs include surgical excision or Mohs micrographic surgery (MMS) [13,14]. MMS is a precise, tissue-sparing method of removing skin cancer that allows for microscopic evaluation of the entire tumor margin [15]. In a network meta-analysis of 40 randomized trials and 5 nonrandomized studies, MMS had a 3.8% rate of recurrence, which was found to be similar to recurrence rates following excision (3.3%), curettage and diathermy (5.9%), and external-beam radiation therapy (3.2%) [16]. Importantly, follow-up duration of the included studies ranged from 1 month to 10 years, and patients included in the studies in this meta-analysis were largely older adults (mean age range 63–66 years), were mostly male (median 61%), and had primarily histologically low-risk superficial or nodular BCC with a mean lesion diameter range of 5–13 mm.

Radiation therapy is an option for patients who are poor surgical candidates or for those who prefer a nonsurgical approach in cosmetically sensitive areas [13,14]. Image-guided superficial radiation therapy (IGSRT), a newer treatment modality cleared by the United States Food and Drug Administration in 2015, uses an integrated high-resolution dermal ultrasound technology to improve lesion visualization. This allows for more precise radiation targeting owing to a more accurate assessment of the tumor depth and width, allowing clinicians to provide adaptive radiation treatment planning. In IGSRT, an ultrasound set to a frequency of ~22 MHz, the optimal frequency for evaluating a skin layer with a depth of 0–6 mm, is used to determine the extent of the lesion beyond clinical visibility [17]. IGSRT has demonstrated a 99.3% rate of local tumor control in 2917 NMSC lesions with a median 14.5 month follow-up [17]. Similarly, an analysis of 1899 NMSC lesions treated with IGSRT found a 99.7% absolute lesion control with an average of 7.5 weeks of treatment, a stable control rate of 99.6% with follow-up >12 months, and a 60-month local control of 99.4% [18]. Retrospective cohort studies have also shown that IGSRT for early-stage NMSCs is clinically equivalent to MMS and statistically significantly superior to traditional non-image-guided SRT and other radiation therapy technologies at 2 years’ follow-up [19,20]. Specifically, in comparison with traditional SRT, IGSRT has demonstrated statistically superior 2-year recurrence rates (0.7% overall, 1.1% for BCC, 0.8% for SCC, and 0.0% for SCCIS) in a retrospective cohort study of 2880 lesions [20]. Furthermore, meta-analyses of two studies evaluating IGSRT and four studies evaluating traditional SRT found that local control of early-stage, high- and low-risk NMSCs was statistically superior with IGSRT compared with traditional SRT overall and in all cancer subtypes when stratified by histology [21,22].

There is now a need for larger cohort studies to evaluate freedom from recurrence following IGSRT stratified by patient and disease characteristics, such as patient age, sex, socioeconomic status, tumor location, and lesion histology. This will aid in identifying the most suitable tumor types and subtypes for IGSRT. Therefore, the objective of this large retrospective cohort study was to determine the effect of histology (BCC, SCC, or SCCIS) and NMSC subtypes on the freedom from recurrence rates in patients with NMSC treated with IGSRT.

## 2. Materials and Methods

### 2.1. IGSRT Treatment Methodology and Energy/Dose Selection Process

The treatment methodology has been previously described in detail [17,18] and follows a general guideline (the Ladd–Yu protocol) [17] for treatment dose, energy, fractionation, and therapeutic biologic effect. A standardized protocol with a total of ~20 fractions using single energy or a sequential combination of 50 kVp, 70 kVp, or 100 kVp energy X-ray treatment is generally delivered 2–4 times per week with pre-treatment. Daily high-resolution dermal ultrasound (HRDUS) prior to “beam-on” is performed to assess/confirm tumor configuration/location and detect changes that may indicate a prescription change as necessary, with adaptive radiation treatment planning. HRDUS is also performed during initial simulation for treatment-planning purposes and at follow-up evaluations after treatment course completion to evaluate response.

### 2.2. Tumor Configuration and Depth Determination

HRDUS uses a non-invasive 20–22 MHz ultrasound with a Doppler component probe that is intrinsic to the IGSRT unit (Sensus SRT-100 Vision), which allows visualization of 0–10 mm into the skin structure, including visualization of the epithelium, papillary layer, and sometimes down to the reticular layer depending on anatomic location and skin thickness. This high-resolution/high-frequency ultrasound allows clear visualization of normal skin anatomy and the disrupting tumor, which occupies a black space and is hypoechoic without Doppler color speckles, and allows for the precise visualization, measurement, and capture of the exact depth of penetration, allowing the clinician to perform adaptive radiation therapy planning during a course of care, analogous to how surgeons can assess efficacy and adapt their approach between individual stages of resection during MMS. The width and configuration of the tumor can also be easily discerned with HRDUS and is integral to localization and treatment planning, reducing the risk of anatomical miss and misadministration.

### 2.3. Data Collection

Data collection followed a similar process as described in published studies [17,18]. IGSRT treatment records of over 11,000 patients with 20,069 NMSC lesions treated at multiple institutions across the continental United States between 2016 and 2023 were retrospectively gathered. Shave biopsies were typically used to confirm initial NMSC histological diagnoses. Recurrences were identified at follow-up visits by the biopsy of suspicious lesions found via clinical exam, HRDUS, and/or dermoscopy. Exclusion criteria include cases that were not nonmelanoma skin cancers (i.e., keloids); missing pertinent documentation (i.e., treatment chart and simulation stats like time, dose, and fractionation); stage 3 tumors with deep invasion, cortical erosion, or perineural invasion; and stage 4 tumors. Lesions that were not treated with a curative dose were included in this intention-to-treat analysis. Patient characteristics (age, sex, and skin cancer history), tumor characteristics (tumor site, histopathologic type and subtype, stage, and depth on ultrasound), treatment parameters (time, dose, and fractionation (TDF); energy; applicator and shield sizes; number of treatments), and treatment outcomes (side effect types and severity, recurrences for these lesions) were extracted manually and accessed electronically from written and electronic medical records (EMRs) for all institutions. Additional data from the EMRs, including race, ethnicity, past medical history, past surgical history, medications, follow-up dates, and mortality status/expiratory dates were collected with algorithmic programming conducted by Sympto Health, Inc. (Santa Clara, CA, USA).

### 2.4. Statistical Analysis

Detailed logs of NMSC recurrences were maintained by the dermatology practices’ radiation therapists. These recurrence logs were used to quantify recurrence events. Freedom from recurrence was estimated using the Kaplan–Meier method. Groups were compared with respect to freedom from recurrence using the log-rank test.

### 2.5. Ethics

The ethics committee/Institutional Review Board (IRB) of WIRB-Copernicus Group (WCG™) waived ethical approval for this work. The dataset was de-identified prior to analysis and all data personnel adhered to the Health Insurance Portability and Accountability Act (HIPAA) and ethical standards to protect patient information.

## 3. Results

### 3.1. Patient and Disease Characteristics

Demographic and disease characteristics are summarized in Table 1. A total of 19,988 lesions were included. Patients were mostly male (61.7%) and aged ≥ 65 years (84.2%); the median age was 74.9 years. Most lesions were located on the head or neck (63.7%), which are known high-risk BCC and SCC locations [1], and most were categorized as stage 0 (23.4%) or stage 1 (65.7%). With regards to histology, lesions were diagnosed as BCC in 49.5% of lesions, SCC in 26.4%, SCCIS in 23.2%, and ≥2 NMSCs in 1.0%.

### 3.2. Freedom from Recurrence Rates by Histology

**Figure 3:**
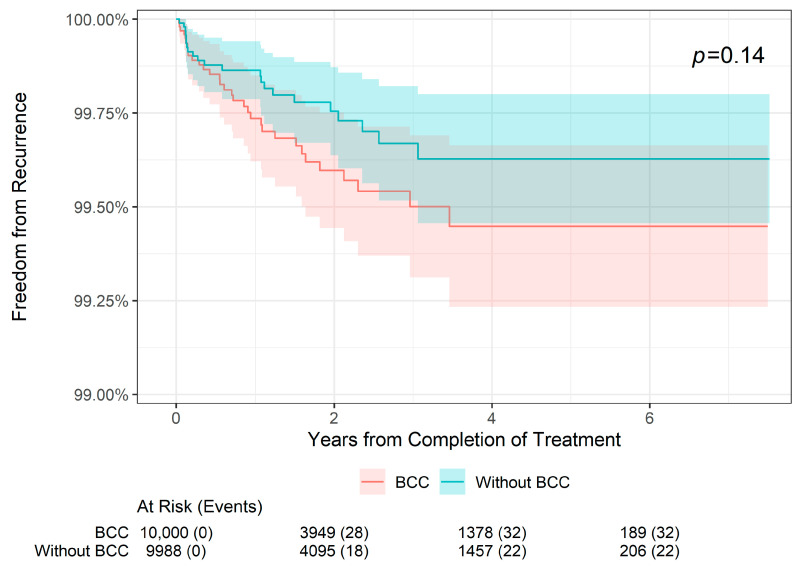
Freedom from recurrence over time of non-melanoma skin cancers treated with image-guided superficial radiation therapy in patients with basal cell carcinoma versus non-basal cell carcinoma skin cancers.

**Figure 4:**
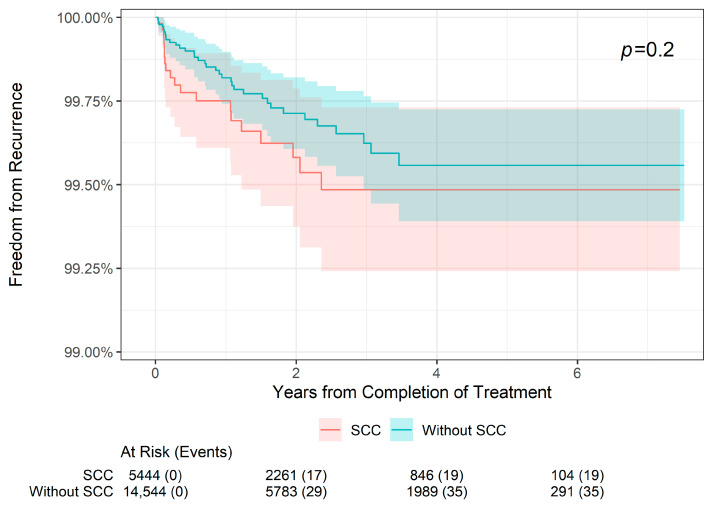
Freedom from recurrence over time of non-melanoma skin cancers treated with image-guided superficial radiation therapy in patients with squamous cell carcinoma versus non-squamous cell carcinoma skin cancers.

**Figure 5:**
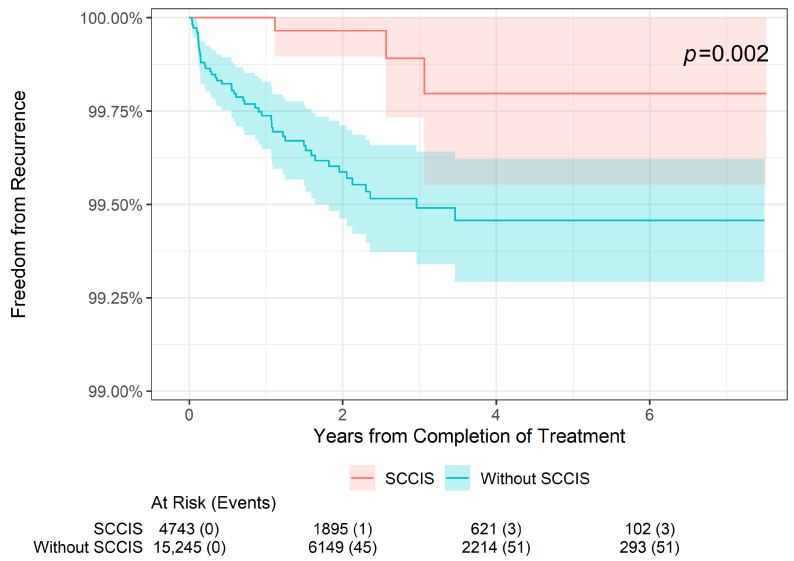
Freedom from recurrence over time of non-melanoma skin cancers treated with image-guided superficial radiation therapy in patients with squamous cell carcinoma in situ versus non-squamous cell carcinoma in situ skin cancers.

The 2-, 4-, and 6-year recurrence and freedom from recurrence rates are presented by histology in Table 2. In BCC lesions (*n* = 9928), the 2-year freedom from recurrence rate was 99.60% compared with 99.75% in non-BCC lesions (i.e., SCC or SCCIS). The 4-year freedom from recurrence rate was 99.45% compared with 99.63% in non-BCC lesions, and the 6-year freedom from recurrence rate was 99.45% compared with 99.63% in non-BCC lesions. These differences were not statistically different (*p* = 0.14; Figure 3). Likewise, in SCC lesions (*n* = 5294), the 2-year freedom from recurrence rate was 99.58% compared with 99.71% in non-SCC lesions, the 4-year freedom from recurrence rate was 99.49% compared with 99.56% in non-SCC lesions, and the 6-year freedom from recurrence rate was 99.49% compared with 99.56% in non-SCC lesions; this was also not statistically significant (*p* = 0.2; Figure 4). However, for SCCIS lesions (n = 4648), the 2-, 4-, and 6-year freedom from recurrence rates were slightly higher than in non-SCCIS lesions, and this difference was statistically significant (*p* = 0.002; Figure 5), which is concordant with the lower stage.

### 3.3. Freedom from Recurrence Rates by Histologic Subtype

While these histologic groupings are relatively homogeneous, several specific histologic subtypes bear individual assessment (Table 3). The most common BCC subtype was nodular, with 4699 lesions, and they experienced 2-, 4-, and 6-year freedom from recurrence rates of 99.53%, 99.29%, and 99.29%, respectively. Similarly, the 1335 well-differentiated (WD) SCC lesions had 2-, 4-, and 6-year freedom from recurrence rates of 99.78% each. These subgroup results did not differ significantly from the larger groupings.

**Figure 6:**
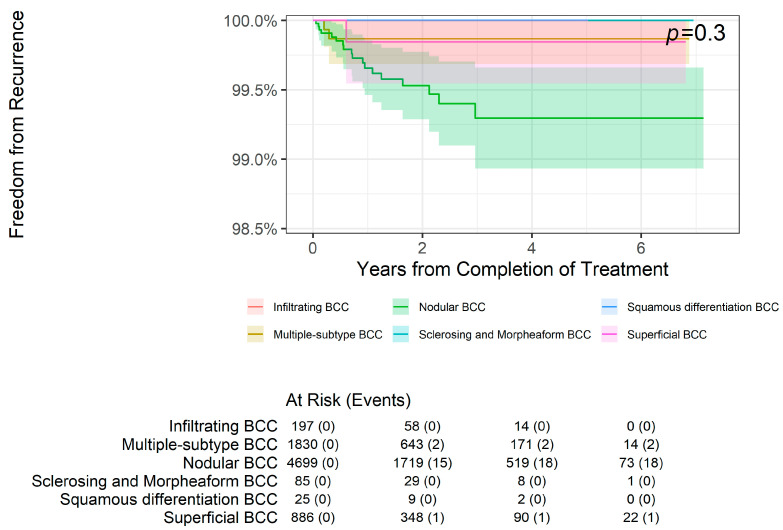
Freedom from recurrence over time of basal cell carcinoma subtypes treated with image-guided superficial radiation therapy.

**Figure 7:**
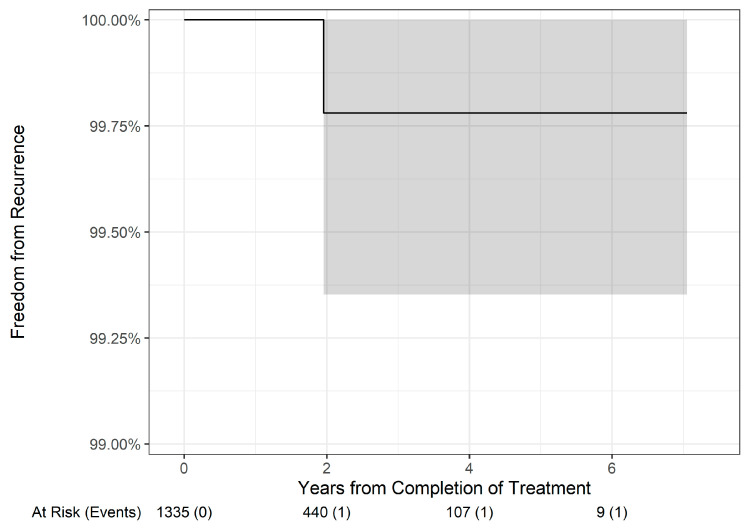
Freedom from recurrence over time of well-differentiated squamous cell carcinoma treated with image-guided superficial radiation therapy.

BCC subtypes (*p* = 0.3, Figure 6) or WD SCC subtype (Figure 7) lesions did not have statistically different overall freedom from recurrence rates.

### 3.4. Example Cases

A few cases of treatment results following IGSRT for NMSCs are highlighted below. Cases demonstrating complete responses and a recurrence are included. All patients had no prior treatment history for their NMSCs undergoing IGSRT.

**Figure 8:**
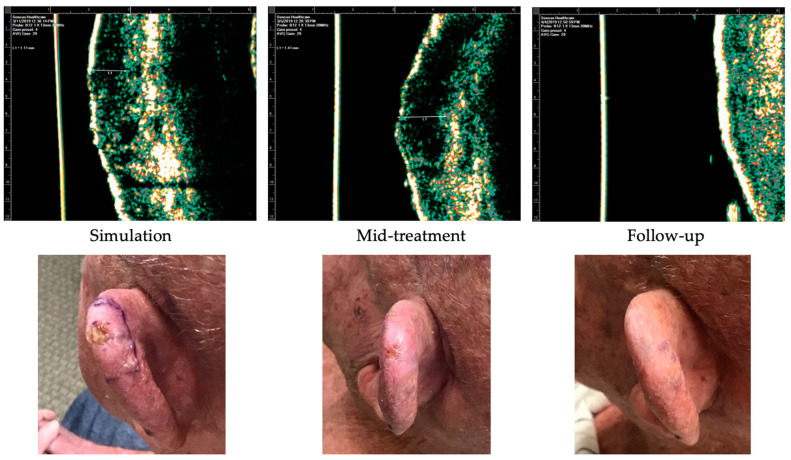
Case 1. Complete response of nodular basal cell carcinoma to IGSRT. Top panels demonstrate the ultrasound images of the IGSRT device before treatment (simulation), mid-treatment, and at final follow-up. The bottom panels demonstrate the clinical response at these same time points.

Case 1 (Figure 8) is of an 81-year-old male who presented with a 0.5 cm nodular BCC on the left superior helix. He received a total dose of 55 Gy delivered in 20 fractions distributed in 3 fractions/week. The most severe side effects were a Radiation Therapy Oncology Group (RTOG) toxicity grade of 2. At a 4-month follow-up, the patient had no clinical evidence of disease.

**Figure 9:**
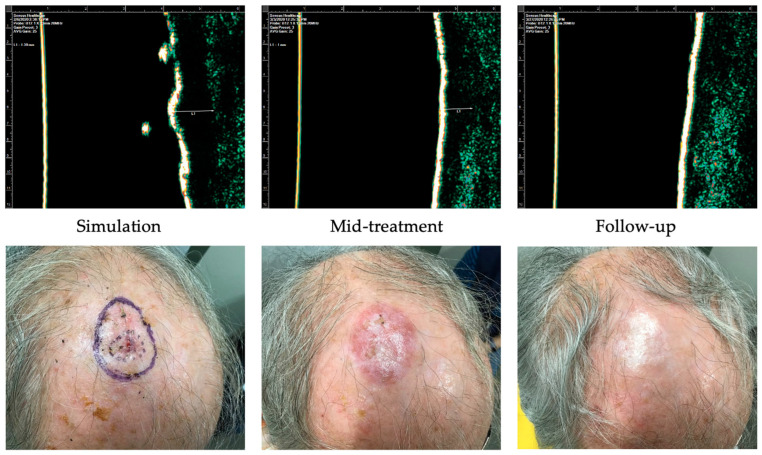
Case 2. Complete response of squamous cell carcinoma to IGSRT. Top panels demonstrate the ultrasound images of the IGSRT device before treatment (simulation), mid-treatment, and at final follow-up. The bottom panels demonstrate the clinical response at these same time points.

Case 2 (Figure 9) is of an 83-year-old male with past medical history significant for diabetes mellitus and hypertension who presented with a 1.8 cm SCC on the scalp. He received a total dose of 54 Gy delivered in 20 fractions distributed in 3 fractions/week. The worst radiation toxicity was graded RTOG 2. At a 3-week follow-up, the patient had no clinical evidence of disease.

**Figure 10:**
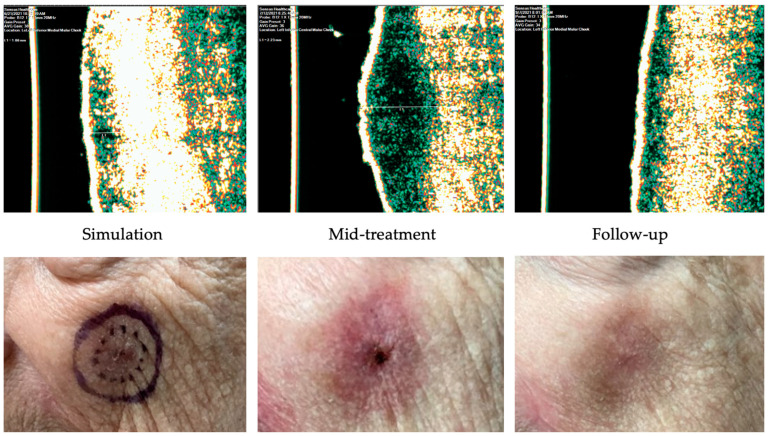
Recurrence of nodular basal cell carcinoma after IGSRT treatment. Top panels demonstrate the ultrasound images of the IGSRT device before treatment (simulation), mid treatment, and at final follow-up. The bottom panels demonstrate the clinical response at these same time points.

Case 3 (Figure 10) is of an 85-year-old female with past medical history significant for hypertension and dementia who presented with a 1.4 cm nodular BCC on the left malar cheek. She received a total dose of 55 Gy delivered in 20 fractions distributed in 3 fractions/week. The worst radiation toxicity was graded RTOG 3. Figure 10 demonstrates the findings at a 4-week follow-up. This site was biopsied at 16 weeks post-IGSRT for concern of recurrence, and a superficial/nodular BCC was identified. The patient was referred to Mohs surgery for further treatment.

## 4. Discussion

The major finding from this intention-to-treat analysis of a large retrospective cohort study is that the effects of IGSRT on invasive NMSCs are not significantly impacted by tumor histology. Freedom from recurrence rates at 2, 4, and 6 years did not differ significantly between BCC vs. non-BCC or SCC vs. non-SCC lesions, suggesting that IGSRT is an equally viable option for either histology. A variety of BCC histologic subtypes and well-differentiated SCCs also had similar freedom from recurrence rates. In contrast, and as was expected, freedom from recurrence rates were slightly higher in SCCIS lesions compared with invasive disease at 2, 4, and 6 years. Finally, freedom from recurrence was not affected by BCC or SCC histological subtype.

These findings demonstrate that IGSRT is a viable therapeutic option for NMSC regardless of histology type or subtype and are supportive of previous research that found that IGSRT is a safe, well-tolerated therapy that demonstrates excellent local tumor control and absolute lesion control [17]. These results also confirm the previously reported superior recurrence rates compared with traditional non-image-guided SRT [19,20].

IGSRT has several advantages as a treatment modality, including being well-tolerated, relatively quick to administer, and suitable for patients who have contraindications for surgery or who refuse surgery. Furthermore, IGSRT preserves function and provides favorable cosmetic outcomes, thereby supporting the primary goals of skin cancer treatment: “the complete removal of the tumor and the maximal preservation of function and cosmesis” [13,14,19].

Superficial radiation therapy is not limited to IGSRT technology. Brachytherapy is contact interventional radiotherapy. This treatment modality offers an over 95% 5-year local control for NMSCs [23]. Typically, treatment is limited to tumors less than 5 mm in depth, but a recent study showed that a multilayer catheter arrangement at various skin-to-catheter distances offers therapeutic windows that encompass thicker tumors while still maintaining favorable toxicity profiles to healthy skin [24].

While early-stage NMSCs are effectively managed by a dermatologist, advanced-stage disease warrants a multidisciplinary care team. Medical oncologists may be needed for directing immunotherapy treatments, surgical oncologists and otolaryngologists might be necessary for resection of extensive disease, and radiation oncologists may deliver radiation in addition to or instead of surgery. Additionally, pathologists fill a key role in the diagnosis of skin cancer by microscopic review of biopsies, and radiologists may be needed for staging scans (CT and/or PET scans). The potential for multidisciplinary management of NMSC highlights the importance of increasing awareness of IGSRT beyond the field of dermatology. This will improve the understanding of a patient’s skin cancer treatment history within multidisciplinary care teams and perhaps facilitate the exploration of IGSRT as part of combination treatments in more advanced NMSC tumors.

Notably, in addition to improving therapeutic accuracy with lesion margin and depth assessment during treatment, the use of HRDUS in IGSRT offers diagnostic benefits. In a study of 323 NMSC lesions, 33% of pre-treatment biopsies under-called the aggressiveness of the NMSCs and 17% over-called it [25]. This demonstrates an opportunity to improve lesion assessment via HRDUS. HRDUS provides real-time evaluation of cancers undergoing treatment. It can assess local aggressiveness by the identification of tumor invasion of deeper structures like soft tissues, cartilage, and adjacent bone, vascularity patterns, and possible microsatellites [26]. Furthermore, HRDUS can be used in post-treatment follow-up visits to identify potential recurrences, which are confirmed by biopsy.

Dermoscopy and line-field confocal optical coherence tomography (LC-OCT) are also useful tools for assessing the margins of NMSCs and therefore improving biopsy selections and treatment planning. Dermoscopy is the use of a high-powered magnification tool with a light source, a dermatoscope, to aid in the diagnosis of skin lesions. The diagnostic accuracy of dermoscopy for BCC is high. The sensitivity and specificity in BCC diagnosis are 91% and 95%, respectively [27]. For SCC, dermatoscopic diagnostic accuracy is lower but still notable, with 79% sensitivity and 87% specificity [28]. A meta-analysis on the rates of incomplete surgical excision of NMSCs with dermoscopy margin evaluation versus without found that dermoscopy caried a 0.29 odds ratio, which supports improved margin assessment with a dermatoscope [29]. Line-field confocal optical coherence tomography (LC-OCT) is a device that produces high-resolution 3D images of skin lesions via a two-beam interference microscope, a laser (light source), and a line camera (photodetector) [30]. For the diagnosis of NMSCs and melanoma, the specificity and sensitivity of LC-OCT is 91% and 87%, respectively [31]. A case–control study compared preoperative margin mapping by LC-OCT versus traditional clinical margin assessment (control) for BCCs undergoing MMS. The LC-OCT group was significantly less likely to have more than one MMS stage resected than the control group, indicating superior margin assessment to clinical/dermoscopic margin assessment [32].

The most significant limitation of this study is its retrospective design, which allows for the analysis of correlation but not causation. Future prospective trials could possibly improve the quality of the evidence available regarding the impact of individual patient and disease characteristics on the effects of IGSRT on freedom from recurrence in patients with NMSC.

Further retrospective analyses by other specific demographic and disease characteristics, such as age, tumor location, socioeconomic status, and comorbidities, are needed to gather insights into the potential effects of these characteristics on disease recurrence and to characterize the patient populations that might gain the most benefit from IGSRT. Additionally, further work to characterize personalized treatment strategies, including genomic [33,34] and imaging [35] methods, could facilitate better patient selection and more individualized radiation treatment plans, including plans for treatment with IGSRT.

## 5. Conclusions

In summary, this is the first large retrospective cohort study of 20,069 NMSC lesions treated with IGSRT to evaluate and compare freedom from recurrence by tumor histologic type. Overall, this study found that freedom from recurrence rates do not vary significantly among BCC or SCC lesions (or their subtypes) but are improved with SCCIS. In combination with previous findings demonstrating the safety profile and efficacy of IGSRT as well as cohort studies indicating the superiority of IGSRT over SRT, these results further suggest that IGSRT is a viable first-line therapeutic option for patients diagnosed with early-stage NMSCs, particularly for those who cannot tolerate or choose not to undergo surgical removal, regardless of their histology. It is important to offer patients as many effective treatment options as possible, and the results of this study support the use of IGSRT in a range of NMSC types.

## Figures and Tables

**Table 1:** Patient and disease characteristics.

Characteristic	All Lesions (*n* = 19,988)	BCC (*n* = 9885)	SCC (*n* = 5270)	SCCIS (*n* = 4635)	≥2 NMSC (*n* = 198)
Age, *n* (%)					
<65 Years	3152 (15.8)	2042 (20.7)	547 (10.4)	545 (11.8)	18 (9.1)
≥65 Years	16,911 (84.2)	7843 (79.3)	4723 (89.6)	4090 (88.2)	180 (90.9)
Sex, *n* (%)					
Female	7652 (38.3)	3895 (39.4)	1925 (36.5)	1766 (38.1)	66 (33.3)
Male	12,324 (61.7)	5985 (60.6)	3341 (63.4)	2866 (61.9)	132 (66.7)
Missing	12	5	4	3	0
Size, cm, median (IQR)	1.0 (0.9, 1.6)	1.0 (0.8, 1.6)	1.2 (1.0, 2.0)	1.0 (1.0, 1.5)	1.5 (1.0, 2.0)
Missing	354	134	88	129	3
TDF, median (IQR)	96.0 (93.0, 98.0)	96.0 (94.0, 98.0)	96.0 (93.0, 98.0)	96.0 (93.0, 97.0)	97.0 (95.0, 99.0)
Missing	255	131	69	51	4
Energy, *n* (%)					
100 kV	3312 (16.6)	1908 (19.3)	1008 (19.1)	335 (7.2)	61 (30.8)
50 kV	5376 (26.9)	2365 (23.9)	1336 (25.4)	1645 (35.5)	30 (15.2)
70 kV	11,105 (55.6)	5537 (56.0)	2852 (54.1)	2609 (56.3)	107 (54.0)
Other	190 (1.0)	73 (0.7)	72 (1.4)	45 (1.0)	0 (0.0)
Missing	5	2	2	1	0
Tumor Location, *n* (%)					
Head/neck	12,728 (63.7)	7098 (71.8)	2784 (52.8)	2693 (58.1)	153 (77.3)
Ear	1692 (8.5)	861 (8.7)	466 (8.8)	346 (7.5)	19 (9.6)
Scalp	1289 (6.4)	271 (2.7)	435 (8.3)	555 (12.0)	28 (14.1)
Forehead	1807 (9.0)	914 (9.2)	395 (7.5)	477 (10.3)	21 (10.6)
Temple	607 (3.0)	306 (3.1)	145 (2.8)	144 (3.1)	12 (6.1)
Orbit/eyelid	119 (0.6)	90 (0.9)	13 (0.2)	16 (0.3)	0 (0.0)
Nose	3460 (17.3)	2663 (26.9)	399 (7.6)	361 (7.8)	37 (18.6)
Cheek	2956 (14.8)	1439 (14.6)	769 (14.6)	715 (15.4)	33 (16.7)
Mucosal lip	51 (0.3)	16 (0.2)	26 (0.5)	9 (0.2)	0 (0.0)
Chin/mandible	149 (0.7)	112 (1.1)	26 (0.5)	11 (0.2)	0 (0.0)
Neck	760 (3.8)	460 (4.7)	138 (2.6)	155 (3.3)	9 (4.5)
Extremities	4125 (20.6)	1080 (10.9)	1791 (34.0)	1228 (26.5)	26 (13.1)
Shoulder	468 (2.3)	321 (3.2)	69 (1.3)	76 (1.6)	2 (1.0)
Trunk	817 (4.1)	528 (5.3)	126 (2.4)	157 (3.4)	6 (3.0)
Chest	530 (2.7)	275 (2.8)	127 (2.4)	125 (2.7)	3 (1.5)
Back	793 (4.0)	597 (6.0)	84 (1.6)	107 (2.3)	4 (2.0)
Stage, *n* (%)					
0	4635 (23.4)	0 (0.0)	0 (0.0)	4635 (100.0)	0 (0.0)
1	12,996 (65.7)	8436 (86.4)	4410 85.0)	0 (0.0)	150 (76.9)
2	1903 (9.6)	1176 (12.1)	698 (13.5)	0 (0.0)	29 (14.9)
3	243 (1.2)	147 (1.5)	80 (1.5)	0 (0.0)	16 (8.2)
Missing	211	126	82	0	3

Abbreviations: BCC, basal cell carcinoma; IQR, interquartile range; NMSC, non-melanoma skin cancer; SCC, squamous cell carcinoma; SCCIS, squamous cell carcinoma in situ. Staging is based on the American Joint Committee on Cancer 8th edition non-Merkel NMSC classification system.

**Table 2:** Freedom from recurrence rates by histology.

Histology	2-Year Freedom from Recurrence	4-Year Freedom from Recurrence	6-Year Freedom from Recurrence
BCC			
At risk, *n* (events)	3949 (28)	1378 (32)	189 (32)
Freedom from recurrence, %	99.60	99.45	99.45
Without BCC			
At risk, *n* (events)	4095 (18)	1457 (22)	206 (22)
Freedom from recurrence, %	99.75	99.63	99.63
SCC			
At risk, *n* (events)	2261 (17)	846 (19)	104 (19)
Freedom from recurrence, %	99.58	99.49	99.49
Without SCC			
At risk, *n* (events)	5783 (29)	1989 (35)	291 (35)
Freedom from recurrence, %	99.71	99.56	99.56
SCCIS			
At risk, *n* (events)	1895 (1)	621 (3)	102 (3)
Freedom from recurrence, %	99.96	99.80	99.80
Without SCCIS			
At risk, *n* (events)	6149 (45)	2214 (51)	293 (51)
Freedom from recurrence, %	99.59	99.46	99.46

Abbreviations: BCC, basal cell carcinoma; SCC, squamous cell carcinoma; SCCIS, squamous cell carcinoma in-situ. Staging is based on the American Joint Committee on Cancer 8th edition non-Merkel NMSC classification system.

**Table 3:** Freedom from recurrence rates by histologic subtype.

Histologic Subtype	2-Year Freedom from Recurrence	4-Year Freedom from Recurrence	6-Year Freedom from Recurrence
Nodular BCC			
At risk, *n* (events)	1706 (15)	515 (18)	73 (18)
Freedom from recurrence, %	99.53	99.29	99.29
Multiple-subtype BCC			
At risk, *n* (events)	642 (2)	170 (2)	14 (2)
Freedom from recurrence, %	99.87	99.87	99.87
Superficial BCC			
At risk, *n* (events)	346 (1)	90 (1)	22 (1)
Freedom from recurrence, %	99.84	99.84	99.84
Infiltrating BCC			
At risk, *n* (events)	57 (0)	13 (0)	NA (NA)
Freedom from recurrence, %	100.00	100.00	NA
Morpheaform BCC			
At risk, *n* (events)	29 (0)	8 (0)	1 (0)
Freedom from recurrence, %	100.00	100.00	100.00
Squamous differentiation BCC			
At risk, *n* (events)	9 (0)	2 (0)	NA (NA)
Freedom from recurrence, %	100.00	100.00	NA
Well-differentiated SCC			
At risk, *n* (events)	440 (1)	107 (1)	9 (1)
Freedom from recurrence, %	99.78	99.78	99.78

Abbreviations: BCC, basal cell carcinoma; NA, not available; SCC, squamous cell carcinoma.

## Data Availability

Data are not publicly available but are available upon reasonable request from the corresponding author.
